# Computational and Imaging Approaches for Precision Characterization of Bone, Cartilage, and Synovial Biomolecules

**DOI:** 10.3390/jpm15070298

**Published:** 2025-07-09

**Authors:** Rahul Kumar, Kyle Sporn, Vibhav Prabhakar, Ahab Alnemri, Akshay Khanna, Phani Paladugu, Chirag Gowda, Louis Clarkson, Nasif Zaman, Alireza Tavakkoli

**Affiliations:** 1 Department of Biochemistry and Molecular Biology, School of Medicine, University of Miami Miller, 1600 NW 10th Avenue, Miami, FL 33136, USA; gowdachirag24@gmail.com; 2 Norton College of Medicine, Upstate Medical University, Syracuse, NY 13210, USA; spornk@upstate.edu; 3 Department of Biological Sciences, University of Illinois at Chicago, Chicago, IL 60607, USA; vibhavp2@uic.edu; 4 Perelman School of Medicine, University of Pennsylvania, Philadelphia, PA 19104, USA; ahab.alnemri@pennmedicine.upenn.edu; 5 Sidney Kimmel Medical College, Thomas Jefferson University, Philadelphia, PA 19144, USA; aya156@students.jefferson.edu (A.K.); phani.paladugu@students.jefferson.edu (P.P.); 6 Brigham and Women’s Hospital, Harvard Medical School, Boston, MA 02115, USA; 7 School of Medicine, University of Cambridge, Cambridge CB2 1TN, UK; louis.clarkson3@nhs.net; 8 Human-Machine Perception Laboratory, Department of Computer Science, University of Nevada Reno, Reno, NV 89557, USA; zaman@nevada.unr.edu (N.Z.); tavakkol@unr.edu (A.T.)

**Keywords:** joint disease, cartilage, synovium, bone matrix, MRI, quantitative CT, spatial transcriptomics, deep learning, molecular imaging, musculoskeletal biomarker

## Abstract

**Background/Objectives:** Degenerative joint diseases (DJDs) involve intricate molecular disruptions within bone, cartilage, and synovial tissues, often preceding overt radiographic changes. These tissues exhibit complex biomolecular architectures and their degeneration leads to microstructural disorganization and inflammation that are challenging to detect with conventional imaging techniques. This review aims to synthesize recent advances in imaging, computational modeling, and sequencing technologies that enable high-resolution, non-invasive characterization of joint tissue health. **Methods:** We examined advanced modalities including high-resolution MRI (e.g., T1ρ, sodium MRI), quantitative and dual-energy CT (qCT, DECT), and ultrasound elastography, integrating them with radiomics, deep learning, and multi-scale modeling approaches. We also evaluated RNA-seq, spatial transcriptomics, and mass spectrometry-based proteomics for omics-guided imaging biomarker discovery. **Results:** Emerging technologies now permit detailed visualization of proteoglycan content, collagen integrity, mineralization patterns, and inflammatory microenvironments. Computational frameworks ranging from convolutional neural networks to finite element and agent-based models enhance diagnostic granularity. Multi-omics integration links imaging phenotypes to gene and protein expression, enabling predictive modeling of tissue remodeling, risk stratification, and personalized therapy planning. **Conclusions:** The convergence of imaging, AI, and molecular profiling is transforming musculoskeletal diagnostics. These synergistic platforms enable early detection, multi-parametric tissue assessment, and targeted intervention. Widespread clinical integration requires robust data infrastructure, regulatory compliance, and physician education, but offers a pathway toward precision musculoskeletal care.

## 1. Introduction

Bone, cartilage, and synovial tissues contain an extensive biomolecular and regenerative capacity [1]. Bone is made up of a mineralized matrix of hydroxyapatite and type I collagen and is modulated by non-collagenous proteins like osteocalcin, while cartilage relies on type II collagen and aggrecan for viscoelasticity, and synovium regulates joint lubrication through hyaluronan and cytokine interactions [2,3,4]. Degenerative processes disrupt these biomolecular networks and induce micro-level changes like collagen crosslink density alterations and cytokine infiltration [5]. This makes it very difficult for clinicians and researchers alike to precisely characterize degenerative joint diseases, particularly in a non-invasive, rapid method that benefits patients [6]. In fact, joint disease involves collagen degradation, proteoglycan depletion, and synovial inflammation, which are often only detectable at the molecular level [7,8]. Fortunately, recent imaging modalities, computational analytics, and sequencing technologies have transformed this landscape and are increasingly allowing physicians to map joint tissue microenvironments [9,10,11].

MRI and qCT, when combined with computational models and sequencing, can provide multi-parametric insights into these diseases [12]. In this review, we explore these synergies and demonstrate how they can advance musculoskeletal care [13]. In essence, our goal is to provide a comprehensive, technical analysis of these methodologies by synthesizing new and proposed cutting-edge imaging, computational, and sequencing approaches [14]. We specifically explore high-resolution MRI, qCT, DECT, ultrasound elastography, radiomics, deep learning, molecular simulations, and sequencing technologies like RNA-seq and spatial transcriptomics [15,16,17]. We also analyze multimodal data fusion with omics datasets and translational applications, including image-guided profiling [18]. Ultimately, we advocate that by carefully implementing these tools, clinicians can significantly advance day-to-day care for patients affected by joint disease [19]. This review offers a uniquely comprehensive synthesis by integrating imaging modalities, artificial intelligence, and multi-omics technologies for musculoskeletal diagnostics. While prior reviews often focus on a single technique or application, our work seeks to emphasize the convergence of high-resolution imaging with radiomic feature extraction and transcriptomics data to enable personalized assessment of joint disease. It is noteworthy to mention that we organize our discussion around the progressive integration of diagnostic technologies, particularly imaging, computational modeling, and multi-omics, rather than solely by tissue type to reflect how these tools converge in clinical and translational workflows. Section 3 will begin by outlining the molecular architecture of bone, cartilage, and synovial tissues in order to understand how emerging diagnostic tools incorporate these features.

## 2. Methodology

This manuscript is structured as a narrative review synthesizing peer-reviewed literature with a particular focus on studies published between 2010 and 2024. To identify sources, we conducted a targeted literature search using databases such as PubMed, Scopus, and IEEE Xplore: search terms included “musculoskeletal imaging”, “RNA-seq joint disease”, “ultrasound elastography cartilage”, “radiomics osteoarthritis”, and “deep learning synovium”. Both clinical and preclinical studies relevant to imaging, computational modeling, or sequencing of bone, cartilage, and synovial tissues were selected. Studies were grouped by methodology (e.g., high-resolution MRI, qCT, RNA-seq), and their translational relevance was assessed based on validation, data availability, and clinical use cases. Studies focusing solely on basic science without translational application were excluded to maintain clinical and diagnostic focus. Wherever applicable, cited experimental works utilized de-identified datasets which received ethical approval for human subject research. Preclinical studies referenced in this review followed animal research ethics protocols as stated in their respective publications. This review does not present original research requiring ethical clearance but draws from ethically conducted and published sources.

## 3. Biomolecular Architecture of Joint Tissues

### 3.1. Bone, Cartilage, and Synovium Composition and Molecular Interactions

As mentioned prior, the mineralized matrix of the bone is primarily composed of hydroxyapatite and type I collagen [20]. These matrix components provide mechanical strength while osteocalcin and osteopontin mediate calcium ion coordination [21]. Cartilage, on the other hand, is composed of type II collagen and aggrecan and maintains viscoelastic properties through proteoglycan-water interactions, which are stabilized by hydrogen bonding networks [22,23]. Synovium, which is rich in hyaluronan and lubricin, regulates joint lubrication, while cytokines like IL-6 and TNF-α modulate inflammatory cascades [24,25]. These biomolecular networks are considered to be dynamic as matrix metalloproteinases (MMPs) are known to cause degradation in pathological states [26].

The structural organization of these tissues influences their biomechanical properties [27]. In bone, collagen fibrils align in lamellar patterns, with hydroxyapatite crystals nucleating along fibril surfaces through electrostatic interactions [28]. The cartilage collagen network forms a porous matrix that entraps aggrecan molecules that regulate water retention through osmotic pressure [29]. Synovial fluid viscosity is dependent on hyaluronan molecular weight and is modulated by shear-induced conformational changes [30].

### 3.2. Implications for Imaging and Computer Modeling

Understanding these molecular interactions is key to determining what impact disease-related abnormalities, like collagen disorganization or cytokine upregulation, has on joint health [31]. Advanced imaging modalities like T1ρ MRI and DECT can capture these biomolecular signatures by probing water dynamics, GAG content, and mineralization patterns [32,33]. Another invasive tool could be computational models like molecular dynamics (MD) simulations, which clinicians can use to simulate collagen crosslink stability or osteocalcin-hydroxyapatite binding based on patient age, weight, and other relevant health measures [34]. Sequencing technologies also have a potential role here. RNA-seq can identify gene expression profiles, such as MMP upregulation, and correlate this with imaging-derived biomarkers [35]. By performing this kind of multivariate analysis, clinicians are conducting multi-parametric healthcare, whereby they can make clinical decisions with both molecular and tissue-level health information [36]. The ultimate goal in blending imaging, computational, and sequencing approaches is to map biomolecular changes to clinical phenotypes [37]. For instance, tracking collagen crosslink density can inform predicted cartilage repair strategies following a certain treatment [38]. Similarly, if patients have their synovial cytokine environment profiled, physicians can recommend certain anti-inflammatory therapies that best align with their cytokine microenvironment [39]. Spatial transcriptomics can also be used in this context since it further localizes gene expression [40].

## 4. Challenges of Conventional Diagnostics

Despite this emerging molecular understanding, we believe that conventional diagnostic modalities are inherently limited by their design and intended purpose [41]. Radiography relies on X-ray attenuation, which can’t capture subtle cartilage degradation or synovial inflammation [42]. Specifically, an X-ray visualizes gross bone loss without sufficient tissue-specific contrast [43]. Histology can be layered on top of X-ray but can be invasive as it requires ex vivo samples [44]. Histological analysis also is inefficient for dynamic, longitudinal monitoring [45]. Many geriatric patients may have prolonged proteoglycan depletion or cytokine infiltration, for example, and sampling at each visit would not be optimal [46]. Two-dimensional radiography is also inappropriate for visualizing three-dimensional tissue architecture, as it does not cover trabecular microarchitecture and nuanced cartilage thinning, while histological staining, despite visualizing collagen and glycosaminoglycan distribution, suffers from sampling bias and fails to assess biomechanical properties, including tissue stiffness [47,48].

## 5. Advanced Imaging Modalities for Joint Tissue Characterization

To overcome these traditional challenges, advanced imaging modalities provide greater tissue specificity and molecular understanding. The following subsections discuss several of these key modalities.

### 5.1. High-Resolution Magnetic Resonance Imaging (MRI)

**Figure 1:**
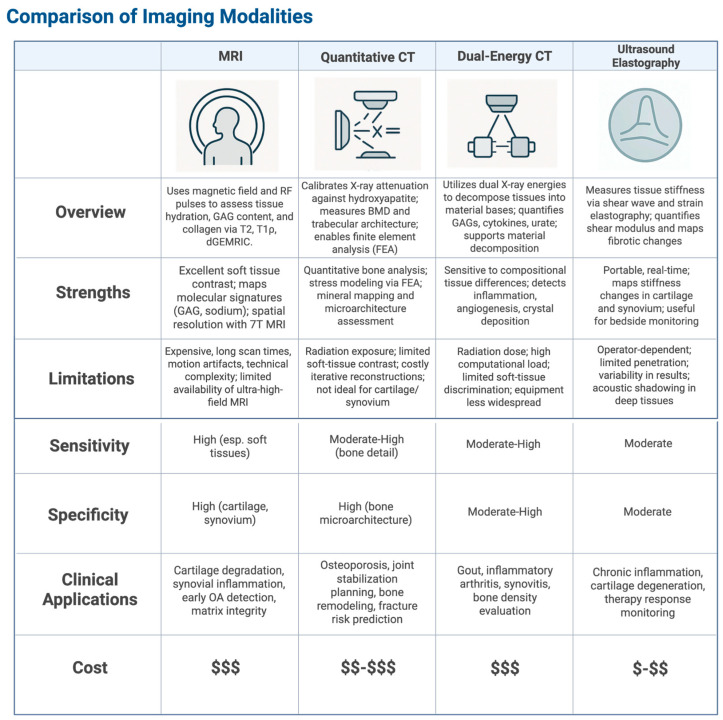
Table comparison of advanced imaging modalities [49,50,51,52,53,54,55,56,57,58,59,60,61,62,63,64,65,66,67,69,70,71,72,73,74,75,76,77,78,79,81,82,83,84,85,86,87,88,89,90,91,93,94,95,96,97,98,99,100,101]. Created in BioRender. Kumar, R. (2025) https://BioRender.com/2tef2wv. accessed on 5 July 2025.

High-resolution Magnetic Resonance Imaging (MRI) uses controlled magnetic field gradients and radiofrequency (RF) pulses to image tissues at a sub-millimeter spatial level [49]. Specialized quantitative techniques, including T2-weighted imaging, assess water content and its interaction with the macromolecular matrix, particularly collagen organization, through measuring spin-spin relaxation times (T2 values, typically 30–80 ms in cartilage), which reflect tissue hydration and matrix integrity (Figure 1) [50]. Researchers can use T1ρ mapping probes (T1 relaxation in the rotating frame) to slow molecular motions and thus quantify proteoglycan concentration by exploiting magnetization decay under specific spin-lock RF pulse conditions [51]. Clinicians can also use delayed Gadolinium-Enhanced MRI of Cartilage (dGEMRIC), which uses anionic gadolinium chelates to map glycosaminoglycan (GAG) distribution [52]. The chelates’ uptake is inversely proportional to GAG content because of this due to anionic charge repulsion, and the resulting spread of GAG molecules can be used to map polysaccharides in the extracellular matrix (ECM) [53].

Interestingly, ultra-high-field MRI (≥7 Tesla) pushes spatial resolution to below 0.3 mm^3^ voxels, which means fine structures like subchondral bone trabeculae and synovial microvasculature can be resolved [54]. Sodium MRI (^23^Na MRI) directly targets sodium nuclei predominantly bound to GAGs, leveraging the quadrupolar nature of ^23^Na and chemical shift imaging to distinguish bound from free sodium [55]. Thus, the bound sodium can be a direct biomarker for cartilage matrix integrity [56].

However, high fidelity does come with its own challenges. High-field systems exacerbate B0 magnetic field inhomogeneities and susceptibility artifacts, particularly at tissue interfaces, which can distort signal intensity and compromise quantification [57]. Technicians can use compressed sensing algorithms to reconstruct images from undersampled k-space data to significantly reduce protracted scan times, and emerging deep learning-based denoising techniques have been shown to enhance image quality [58,59]. Advanced pulse sequences, including 3D spoiled gradient echo (SPGR), are optimized for specific applications like cartilage visualization by maximizing the contrast between articular surfaces and synovial fluid, and quantitative susceptibility mapping (QSM) assesses tissue mineralization by exploiting magnetic susceptibility differences [60,61]. AI-driven segmentation and multi-parametric analysis are increasingly integrated to extract comprehensive phenotypic information from these rich datasets [62]. Nevertheless, physicians face substantial hurdles in translating these advanced MRI capabilities into routine clinical practice. Beyond intrinsic technical demands like precise B0 shimming, prolonged scan durations and susceptibility to motion artifacts must be accounted for [63]. Motion artifacts can be mitigated if technicians use parallel imaging like SENSE and motion correction algorithms like PROPELLER, but this still may be insufficient [64]. Furthermore, ultra-high-field scanners and specialized sequences can drive up costs, especially due to limited supply and recent NIH funding cuts [65]. Unregulated tools may also lead to standardization issues and inter-scanner variability [66]. Nonetheless, the biomolecular information extracted, such as GAG content or water organization, ultimately reflects ongoing gene expression, RNA processing and its regulation that determine tissue composition and function [67]. Pathological changes detected by MRI often stem from dysregulation within these fundamental biological networks, making non-invasive MRI a powerful tool for probing disease mechanisms rooted in molecular and cellular alterations [68].

### 5.2. Quantitative Computed Tomography (qCT)

Quantitative Computed Tomography (qCT) can precisely measure bone mineral density (BMD) by calibrating X-ray attenuation values against standardized hydroxyapatite phantoms, thus achieving Hounsfield unit precision often below 1% (Figure 1) [69]. Orthopedic specialists would stand to benefit from qCT by utilizing it to visualize three-dimensional trabecular microarchitecture and measure values like trabecular thickness, separation, and connectivity density as a result [70]. These are fundamental determinants of bone strength that would otherwise not appear on standard X-ray alone [71].

While basic CT technology is ubiquitous in hospitals, qCT possesses greater capabilities for advancement. As qCT can conduct finite element analysis (FEA), its data may be used to simulate stress distribution under physiological loads, therefore computing von Mises stresses to more precisely estimate the risk of fracture [72]. If clinicians were to add voxel-based morphometry techniques to qCT data through subtle density gradients, they would be able to track bone remodeling patterns [73]. By adjusting for polychromatic X-ray absorption, technical developments like spectral calibration reduce beam-hardening artifacts and guarantee correct BMD readings [74]. Multi-energy qCT acquisition enhances the contrast between mineralized and soft tissues by differentiating materials like hydroxyapatite and collagen based on their distinct attenuation profiles at different X-ray energies [75]. Modern iterative reconstruction algorithms, including model-based iterative reconstruction (MBIR), are routinely employed to reduce image noise and improve edge detection, which sharpens the visualization of fine trabecular boundaries, allowing for more precise quantification [76]. These capabilities make qCT invaluable for assessing bone quality and understanding degenerative changes. For example, in planning joint stabilization procedures like coracoid graft placement for glenoid reconstruction, qCT can map mineralization gradients to inform graft integration [77]. Low-dose qCT techniques reduce radiation exposure, therefore making the modality appropriate for longitudinal bone health monitoring [78]. Although its use in cartilage and synovial assessment is limited by its low capability to produce soft-tissue contrast as compared to MRI, the integration of qCT with computational tools like radiomics can further extract high-dimensional quantitative features. This, in turn, will be able to improve diagnosis precision for bone-related pathologies [79]. The creation of photon-counting detectors holds great promise in increasing spatial resolution and contrast even more, hence extending the uses of qCT in alignment with the focus of precision medicine on strong imaging biomarkers for individualized diagnosis [80].

### 5.3. Dual-Energy Computed Tomography (DECT)

Dual-Energy Computed Tomography (DECT) works by acquiring data from two distinct X-ray energy spectra (e.g., 80 kVp and 140 kVp), and in turn differentiating between tissue compositions based on material-specific attenuation coefficients (Figure 1) [81]. In essence, this means that images can be decomposed into basis material pairs like hydroxyapatite and collagen and subsequently quantified [82]. DECT is an advanced application of CT, but goes a step further by analyzing differences in Compton scattering and photoelectric absorption; in DECT, attenuation coefficients correlate with GAG concentration [83]. Furthermore, DECT can detect cytokine-driven synovial inflammation through enhanced soft-tissue contrast and identify vascular alterations linked to angiogenesis [84].

Photon-counting detectors significantly improve the spectral resolution of DECT by reducing noise and enhancing contrast-to-noise ratios by more accurately resolving energy-specific photon interactions. Virtual monochromatic imaging, alternatively, reconstructs images at optimal single-energy levels (e.g., 70 keV) to minimize beam-hardening artifacts and ensure consistent tissue characterization [85,86]. Material decomposition algorithms, such as two-material or three-material decomposition, precisely quantify fractions of substances like calcium, uric acid, and soft tissue, supporting multi-parametric analysis of degenerative and metabolic joint diseases [87]. Along with changes like IL-6 infiltration in inflamed synovium (where IL-6 levels reflect active gene expression in response to stimuli), DECT’s ability to map biomolecular gradients, such as urate crystal deposition in gout or subtle calcium changes in bone and soft tissues, helps to support earlier and more specific diagnosis [88]. This guides focused therapy approaches ranging from anti-cytokine biologics for inflammatory arthropathies to urate-lowering treatments for gout to bisphosphonates for bone density problems [89]. Integration of AI-driven segmentation guarantees precise identification of areas of interest (ROIs), hence allowing spatially resolved measurement of tissue changes and matching with the requirement for new imaging biomarkers of precision medicine [90]. Though modern iterative reconstruction techniques and dose modulation strategies help to reduce dose requirements while correcting for scatter and improving general image quality, DECT still faces challenges, including the computational intensity of material decomposition methods and concerns regarding radiation exposure [91].

### 5.4. Ultrasound Elastography

Ultrasound elastography offers a non-invasive approach to quantitatively assess tissue biomechanical properties by measuring stiffness through the tracking of acoustic wave propagation. This is done primarily using Shear Wave Elastography (SWE), a prominent technique that quantifies shear moduli often expressed via shear wave velocity (Figure 1) (Equation (1))

(1) $$C_{s}=\sqrt{\frac{E}{3\rho }}$$

Equation (1): Shear Wave Speed (m/s), where *E* is Young’s modulus in kilopascals, and ρ is density in kg/m^3^, as a surrgate for material integrity [92].

In articular cartilage, SWE can discern alterations in collagen crosslink density and organization: qualities intrinsically linked to chondrocyte gene expression, protein synthesis, and post-translational modifications that define the extracellular matrix. This is because shear wave velocities typically increase proportionally with matrix stiffening [93]. Concurrently, strain elastography, though more qualitative due to its reliance on operator-applied compression, can map tissue deformation in structures like the synovium, identifying fibrotic changes characteristic of chronic inflammatory states, which themselves are outcomes of sustained pathogenic signaling and altered gene expression patterns leading to excessive matrix deposition [94]. The utility of high-frequency transducers (>20 MHz) allows for sub-millimeter spatial resolution, resolving microscale variations in elasticity crucial for detecting early pathology [95]. However, SWE’s accuracy relies on accounting for the viscoelastic and anisotropic nature of tissues like cartilage, often necessitating multi-angle acquisitions, while strain elastography’s inherent operator dependency introduces variability, though automated algorithms and standardized measurement protocols aim to improve reproducibility [96]. The integration of artificial intelligence-driven image processing is further enhancing the capacity to detect subtle, spatially-resolved stiffness changes that are pivotal for the early diagnosis of degenerative phenotypes [97]. The portability and real-time capability of ultrasound elastography make it suitable for point-of-care applications, such as bedside monitoring of synovial inflammation or cartilage integrity, thereby informing therapeutic interventions like corticosteroid injections by mapping stiffness gradients [98]. While its penetration depth limits direct assessment of deeper bone structures, surface-based measurements can indirectly probe subchondral bone stiffness [99]. Despite advancements like 3D elastography promising enhanced volumetric resolution, challenges including operator variability, acoustic shadowing in deeper tissues, and the trade-off between resolution and penetration with high-frequency transducers (particularly in obese patients) persist [100]. Crucially, by correlating macroscopic stiffness measurements with molecular data, for instance, from RNA-sequencing profiles of inflammatory or matrix-remodeling genes, elastography contributes to a precision medicine paradigm, linking imaging-derived biomechanical biomarkers to underlying cellular and molecular perturbations for improved diagnostics and guided interventions [101].

### 5.5. Vibroarthrography

**Figure 2:**
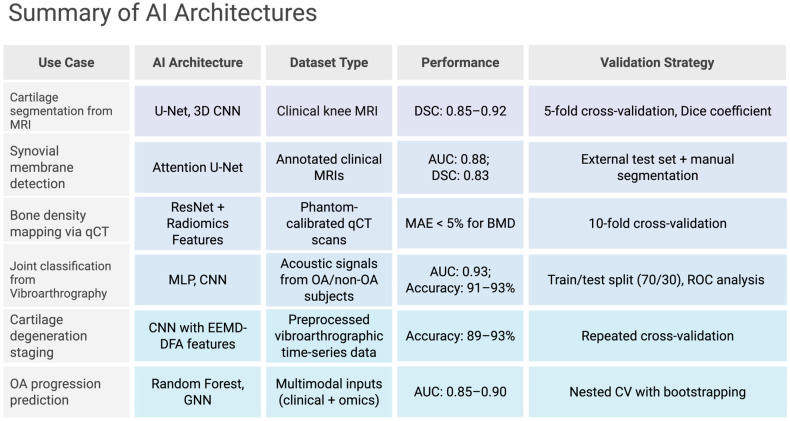
Table summary of pivotal AI Architectures [104,106,127,128,129,130,131,132]. Created in BioRender. Kumar, R. (2025) https://BioRender.com/um658hh (accessed on 5 July 2025).

A more recent and innovative imaging technique that has increasing clinical relevance is vibroarthrography (Figure 2). This non-invasive method assesses the mechanical behavior of articular surfaces through acoustic signal analysis during joint movement, often using accelerometers and piezoelectric sensors placed over the patella and tibial plateau [102]. By capturing joint-generated vibrations, it is able to provide valuable information about cartilage integrity with applications in detecting early degenerative changes [103]. Recent studies have advanced the diagnostic capabilities of vibroarthrography by integrating it with machine learning and advanced signal processing. In one study, acoustic signals were collected from 59 asymptomatic and 40 osteoarthritic knees. Frequency features, particularly high-frequency components normalized for soft tissue interference, were extracted during flexion and extension cycles. When analyzed using a linear support vector machine (SVM), the method achieved a classification specificity of nearly 0.80 and a sensitivity of around 0.75, comparable to existing diagnostic standards [102]. Further refinements using Empirical Mode Decomposition (EEMD) and Detrended Fluctuation Analysis (DFA) enabled multi-scale decomposition and analysis of joint-generated acoustic signals. These techniques isolate intrinsic mode functions (IMFs) from raw signals and quantify scaling properties that correspond to structural irregularities in cartilage. When combined with artificial neural networks (ANNs), specifically a multilayer perceptron (MLP), these signals were found to yield a classification accuracy of 93%, with both sensitivity and specificity also reaching 93% (AUC = 0.942) [104]. This analysis, when performed with recurrence quantification methods, was shown to achieve a classification accuracy of 91.07% with MLP models and 80.36% with radial basis function (RBF) networks [105].

Moreover, the combination of EEMD-DFA feature extraction with convolutional neural networks (CNNs) has been shown to further improve diagnostic precision. The CNN model, trained on decomposed and filtered acoustic data, accurately classified various stages of cartilage degeneration in real-time, proving high translational potential [106]. In aging populations, where early identification of degenerative changes can significantly improve outcomes, vibroarthrography is playing a growing role in OA detection. The low cost, absence of radiation, and potential for point-of-care implementation make vibroarthrography an attractive candidate for widespread screening and longitudinal monitoring [107].

## 6. Computational Frameworks for Biomolecular Analysis

Alongside imaging innovations are computational approaches that enable the extraction of complex biomolecular data. Key among these are radiomic analysis and deep learning (Figure 2).

### 6.1. Radiomic Feature Extraction

Radiomics extracts quantitative features from imaging data to capture texture, intensity, and shape characteristics [108]. Gray-level co-occurrence matrix (GLCM) features, such as entropy and contrast, can quantify collagen disorganization in cartilage, while wavelet-based features detect mineralization heterogeneity in bone [109]. Histogram-based metrics like skewness and kurtosis can measure signal intensity distributions, which clinicians can interpret as correlating with proteoglycan content [110]. All in all, clinicians can use GLCM features with histogram metrics to understand a more holistic picture of degenerative change [111]. In radiomic pipelines, radiologists preprocess images through intensity normalization and denoising, followed by region-of-interest (ROI) segmentation using thresholding or active contour models [112]. Feature extraction employs libraries like Pyradiomics, generating high-dimensional datasets (>1000 features) [113]. Dimensionality reduction via principal component analysis (PCA) or least absolute shrinkage and selection operator (LASSO) mitigates overfitting, selecting features with high variance or predictive power [114]. Increasingly, machine learning models, such as support vector machines, are now classifying degenerative phenotypes based on radiomic signatures [115]. We strongly believe that integrating ML-powered radiomics with imaging modalities can enhance diagnostic accuracy [116]. In MRI, GLCM features correlate with T1ρ relaxation times, reflecting GAG loss, while in qCT, wavelet features quantify trabecular connectivity [117]. These features support non-invasive biomarker discovery, mapping biomolecular changes to tissue-level pathology [118]. However, a major challenge is feature redundancy and computational complexity, meaning initial data has to be cross-validated between radiologists before models can be deployed [119]. However, integrating imaging with RNA-seq profiles and general sequencing data of MMP expression can help correlate radiomic features with molecular pathways, enhancing interpretability [120]. Ultimately, by incorporating these radiomic pipelines, physicians can now obtain multi-parametric biomarkers from imaging data [121].

### 6.2. Deep Learning Pipelines

Deep learning frameworks like convolutional neural networks (CNNs) can help automate feature extraction and segmentation [122]. U-Net architectures, with encoder-decoder structures, are actively being used to segment cartilage and synovium by learning hierarchical features, thus leveraging skip connections to preserve spatial information [123]. These models have been trained on diverse datasets, including clinical MRI scans from patient cohorts such as the Osteoarthritis Initiative (OAI), as well as ex vivo high-field MRI from preclinical models. Pertinent validation strategies include k-fold cross-validation, external test sets, and manual expert segmentations as ground truth. By transfer learning these architectures, developers can adapt pre-trained models (e.g., ResNet, DenseNet) to musculoskeletal datasets [124]. This is particularly beneficial for more rural hospitals and serves to compensate for limited sample sizes [125].

Vibroarthrography signal classification has benefited from deep learning as well. Specifically, ANN-based models and hybrid architectures trained on acoustic joint signals have achieved promising accuracy in distinguishing between healthy and osteoarthritic joints. These models often rely on features extracted through Empirical Mode Decomposition (EMD), Recurrence Quantification Analysis, and similar preprocessing steps to enhance signal interpretability.

Clinicians can also take advantage of Generative adversarial networks (GANs), as GANs can synthesize high-fidelity images and feed them into new model training datasets [126]. For example, conditional GANs can generate trabecular bone patterns, thus improving CNN performance in fracture risk prediction [127]. Adversarial training goes a step further as well and enhances super-resolution, reconstructing sub-voxel details from low-resolution scans via pixel-wise loss minimization [128]. On the biochemical side, researchers can also integrate AlphaFold2 into GAN-trained CNNs to predict protein structures like collagen or osteocalcin [129]. In theory, clinicians can have the following deep learning pipeline: they begin by preprocessing pictures using intensity normalization and artifact correction, then train models on annotated datasets [130]. Post-processing corrects segmentation faults using conditional random fields, which ensures a high rate of anatomical accuracy [131]. Then, this information is integrated with sequencing data like single-cell RNA-seq such that imaging-derived phenotypes can be correlated to gene expression patterns [132]. Despite these advances, clinical adoption remains limited due to variability in training data, lack of standardized evaluation benchmarks, and concerns regarding model interpretability (Figure 2).

## 7. Multimodal Data Fusion and Omics Integration

**Figure 3:**
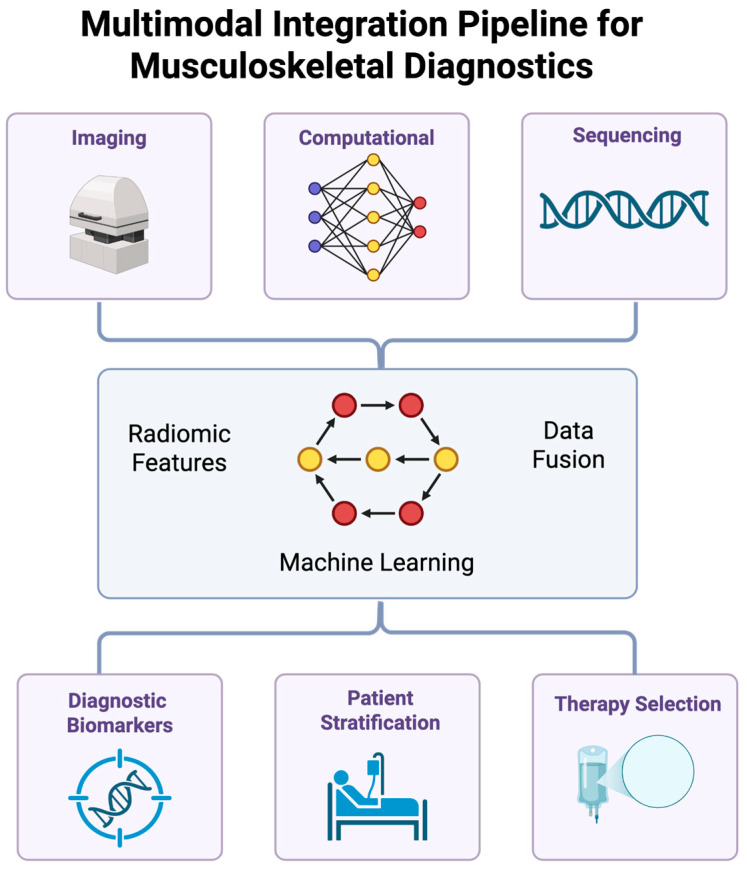
Schematic detailing multimodal data fusion with omics integration. Created in BioRender. Kumar, R. (2025) https://BioRender.com/ck4jzd0 (accessed on 5 July 2025).

These tools, when used in combination with transcriptomic and proteomics data, can enable precision diagnostics. The following subsections will discuss how this integration can serve to reveal novel biomarkers and clarify underlying disease mechanisms (Figure 3).

### 7.1. Transcriptomic Integration

RNA sequencing (RNA-seq) profiles gene expression in joint tissues by identifying inflammatory signatures like IL-6 and TNF-α in synovium [133]. Single-cell RNA-seq resolves cellular heterogeneity, mapping chondrocyte and synoviocyte subpopulations with distinct functional roles [134]. Spatial transcriptomics localizes gene expression across tissue sections, aligning with imaging-derived regions of interest to identify molecular changes, such as collagenase upregulation in cartilage [135]. Imaging-transcriptomic fusion employs canonical correlation analysis (CCA) to correlate MRI-derived radiomic features with gene expression, identifying shared variance in degenerative phenotypes [136].

RNA-seq workflows begin with steps like RNA extraction, library preparation, and sequencing on platforms like Illumina NovaSeq. Then, read alignment using STAR and differential expression analysis with DESeq2 are performed [137]. Imaging data are preprocessed to extract radiomic or biomechanical features and normalized for compatibility with transcriptomic profiles [138]. Multi-view learning integrates these datasets, learning joint representations via deep neural networks [139]. This approach quantifies disease-specific gene networks, guiding targeted interventions like anti-cytokine therapies [140].

Spatial transcriptomics employs techniques like 10x Visium, capturing mRNA from tissue sections with barcoded probes, enabling spatially resolved gene expression mapping [141]. Integration with DECT or MRI identifies regions of biomolecular alteration, such as synovial inflammation, enhancing diagnostic specificity [142]. These tools support precision medicine by correlating molecular and imaging biomarkers, thereby enabling early detection of degenerative changes in musculoskeletal tissues [143].

Challenges to this method include data heterogeneity and batch effects, which necessitate robust normalization and quality control [144]. Computational platforms, like Seurat, streamline single-cell RNA-seq analysis, while spatial transcriptomics tools, such as Space Ranger, ensure accurate gene localization [145]. The integration of transcriptomic data with imaging and computational frameworks aligns with the special issue’s focus on genomics and imaging, delivering personalized diagnostics for musculoskeletal care [146].

### 7.2. Proteomic Integration

Mass spectrometry-based proteomics enables researchers to quantitatively determine the complex protein landscapes and specific post-translational modifications (PTMs), including phosphorylation, glycosylation, and citrullination, within joint tissues, thereby offering molecular insights into pathological mechanisms [147]. Detection of Cartilage Oligomeric Matrix Protein (COMP) fragments by proteomic interrogation signals collagen network degradation, while distinct osteocalcin isoforms or pro-collagen type I N-terminal propeptide (PINP) levels reflect osteoblastic activity and bone matrix turnover [148]. Investigators utilize synovial fluid proteomics to delineate intricate cytokine profiles, where elevated IL-1β, TNF-α, and VEGF concentrations frequently induce inflammatory cascades and angiogenic responses [149]. Fusing proteomic data with imaging biomarkers, like specific protein signatures with MRI-derived cartilage T2 relaxation times or ultrasound elastography-measured synovial stiffness, for example, frequently employs multivariate statistical approaches like partial least squares regression or canonical correlation analysis to maximize the covariance explained between disparate data modalities [150]. Rigorous proteomic workflows initiate with meticulous protein extraction from minute tissue biopsies or biofluids, followed by reduction, alkylation, and enzymatic digestion, typically using trypsin. Then, peptides are subjected to liquid chromatography coupled with tandem mass spectrometry (LC-MS/MS) on high-resolution instruments like Orbitrap series mass spectrometers (e.g., Orbitrap Exploris or Ascend Tribrid); label-free quantification (LFQ) strategies, such as MaxLFQ or intensity-based absolute quantification (iBAQ), or alternatively, data-independent acquisition (DIA-MS) coupled with spectral library searching (e.g., using Spectronaut or Skyline), ensure comprehensive peptide detection and high-throughput quantitative accuracy [151,152]. Subsequent bioinformatic processing pipelines, often orchestrated within environments like MaxQuant, Proteome Discoverer, or custom R/Python scripts, perform peptide identification via database searching (e.g., against UniProt), protein inference, false discovery rate (FDR) control, and sophisticated statistical analysis to identify differentially abundant proteins [153]. These refined protein abundance datasets are then aligned with preprocessed radiomic or biomechanical features extracted from diverse imaging datasets, facilitated by integrative platforms such as MixOmics or bespoke statistical frameworks, to identify robust protein-imaging biomarkers.For instance, elevated MMP-3 activity correlating with reduced cartilage shear wave velocity is used to support personalized risk stratification algorithms [154]. The critical integration with transcriptomic data, for example, RNA-seq profiles detailing inflammatory gene expression signatures, allows for the correlation of protein expression levels with corresponding mRNA abundances, thereby enhancing mechanistic understanding by revealing post-transcriptional regulatory events [155]. Deep proteomic investigation of protein complexes like the UBE2T-FANCL DNA repair machinery can pinpoint specific ubiquitination sites on target substrates, thereby informing the design of precise therapeutic interventions aimed at modulating DNA repair capacity to promote bone regeneration or combat cellular senescence [156]. These multi-layered omics approaches effectively bridge molecular changes with tissue-level manifestations: a cornerstone of precision medicine’s need for identifying detailed and accurate biomarkers [157]. Notwithstanding these advances, challenges pertaining to inherent sample biological variability, the vast dynamic range of protein concentrations in biological matrices, and the need for highly standardized pre-analytical and analytical protocols persist, highlighting the need for continuous methodological refinement and rigorous quality control to ensure data fidelity and inter-laboratory reproducibility [158]. Computational tools and robust bioinformatics pipelines are indispensable for streamlining these complex analytical workflows, ensuring that quantitative proteomics remains a critical, indispensable component for advancing precision musculoskeletal diagnostics through comprehensive omics integration [159].

### 7.3. AI-Augmented Segmentation

AI-augmented segmentation enhances ROI delineation in imaging, which is critical for spatially-resolved biomolecular quantification [160]. Graph-cut algorithms initialize segmentation, refined by CNNs like U-Net, to achieve sub-voxel accuracy [161]. Attention-based models based on transformer architectures prioritize regions of interest, such as cytokine-rich synovial zones, by weighing feature importance [162]. Segmentation resolves trabecular boundaries in bone, enabling precise BMD quantification, while in cartilage, it maps proteoglycan gradients [163].

Segmentation pipelines preprocess images through intensity normalization and artifact correction, followed by model training on annotated datasets [164]. Transfer learning adapts pre-trained models to musculoskeletal imaging, mitigating data scarcity [165]. Post-processing corrects segmentation errors using conditional random fields, which ensures anatomical fidelity [166]. Integration with spatial transcriptomics correlates segmented ROIs with gene expression, enhancing diagnostic specificity [167].

Deep learning models, like those inspired by AlphaFold2, predict tissue-specific protein structures, informing segmentation by providing molecular context [168]. In synovium, attention mechanisms detect inflammatory foci, improving sensitivity for early disease detection [169]. These tools enable quantitative phenotyping, the tracking of degenerative changes like synovial thickening or bone erosion, and the support of longitudinal monitoring in musculoskeletal care [170].

Challenges include computational complexity and dataset bias, requiring robust validation through k-fold cross-validation [171]. Explainable AI frameworks enhance clinical reliability, ensuring transparency in segmentation outputs [172]. AI-augmented segmentation’s ability to process multimodal imaging data aligns with the special issue’s focus on AI-enhanced imaging, delivering precision diagnostics for degenerative joint diseases [173].

### 7.4. Multi-Scale Modeling

Multi-scale modeling offers a paradigm for synthesizing disparate molecular, cellular, and tissue-level data, thereby constructing a holistic understanding for clinicians; for instance, agent-based models (ABMs) dynamically simulate chondrocyte mechanobiological responses, often incorporating molecular dynamics (MD)-derived parameters for collagen fibril mechanics [174]. Concurrently, finite element models (FEM), critically parameterized by quantitative inputs such as qCT-derived bone microarchitecture or ultrasound elastography-derived tissue shear moduli, are employed by engineers and biomechanists to meticulously compute complex stress-strain distributions under diverse loading scenarios, thus providing invaluable predictions of biomechanical integrity and fracture risk [175]. The intricate dynamics of synovial inflammation are elucidated through sophisticated network models, which may manifest as Boolean networks delineating the logical architecture of signaling cascades (e.g., TNF-α/NF-κB pathways), systems of ordinary differential equations (ODEs) quantifying the kinetic rates of specific biomolecular interactions (e.g., JAK-STAT signaling post-IL-6 receptor engagement), or even multi-cellular agent-based simulations [176]. These predictive engines frequently integrate multi-omics datasets, including transcriptomic profiles from RNA-sequencing that reveal cytokine gene expression landscapes and proteomic analyses quantifying protein abundances. This enables them to forecast inflammatory trajectories driven by complex intercellular dialogues dictated by underlying gene regulatory networks [177]. Specialized computational platforms like COMSOL Multiphysics or open-source alternatives such as OpenCMISS facilitate the unification of these heterogeneous, multi-scale inputs, striving for biophysical consistency across vastly different spatiotemporal scales. Hybrid computational frameworks, on the other hand, synergistically coupled differential equation systems for molecular kinetics with finite difference/element methods for continuum-level tissue mechanics and transport phenomena [178]. Rigorous validation of these computational constructs against empirical imaging data, by comparing predicted cartilage deformation patterns with elastography-derived shear moduli or simulated bone adaptation responses with qCT-visualized mineralization changes, for instance, is indispensable: the integration of high-resolution single-cell RNA-sequencing (scRNA-seq) data provides unparalleled cellular-level specificity, detailing heterogeneous cell populations and their distinct transcriptomic signatures, significantly enhancing model granularity [179]. Consequently, this multi-scale modeling paradigm directly informs therapeutic design by guiding the development of targeted MMP inhibitors based on simulated enzyme activity in cartilage, optimizing biomaterial scaffold properties for bone tissue engineering by predicting mineralization kinetics or forecasting synovial inflammatory responses to biologics to aid in rational dosing strategies [180]. Despite these powerful capabilities, there remain substantial challenges pertaining to computational expense, high-performance computing resources, inherent model complexity demanding meticulous parameterization, and the overarching need for robust, multi-faceted validation against diverse experimental and clinical datasets. As a result, the search for hybrid modeling approaches that judiciously blend deterministic and stochastic methodologies to balance predictive accuracy with computational tractability continues [181].

## 8. Translational Applications in Musculoskeletal Care

### 8.1. Image-Guided Molecular Profiling

Image-guided molecular profiling represents a sophisticated convergence of advanced diagnostic imaging and targeted therapeutic intervention, enabling physicians to leverage precise, patient-specific biomolecular insights for optimizing treatment efficacy in musculoskeletal disorders [182]. For instance, delayed Gadolinium-Enhanced MRI of Cartilage (dGEMRIC) meticulously maps cartilage glycosaminoglycan (GAG) content, which directly reflects chondrocyte biosynthetic activity governed by intricate gene expression programs. This enables researchers to visualize proteoglycan distribution, aiding the development of regenerative strategies such as autologous chondrocyte implantation or stem cell injections [183]. Similarly, quantitative Computed Tomography (qCT) provides high-fidelity quantification of bone microarchitecture and mineral density, both of which are essential data for informing graft sizing and placement in complex joint stabilization procedures like coracoid transfer for glenoid reconstruction, where successful osseointegration is dependent on matching graft properties to host bone characteristics that are themselves outcomes of regulated cellular processes [184]. Dual-Energy CT (DECT) extends these capabilities by detecting and mapping specific molecular signatures of synovial inflammation, such as increased vascularity or even the distribution of pro-inflammatory cytokines like Interleukin-6 (IL-6), whose localized expression is a hallmark of active inflammatory pathways, thus guiding the targeted administration of anti-cytokine therapies [185]. These innovative systems increasingly integrate imaging modalities with powerful computational models; sophisticated profiling workflows employ radiomics to extract sub-visual quantitative features that encapsulate biomolecular signatures, while machine learning algorithms, trained on extensive multimodal datasets, are developed to predict therapeutic responses and long-term outcomes [186]. The drive for real-time intraoperative decision support, particularly in interventions like precise graft placement during bone repair or navigation in minimally invasive spine surgery (where techniques like uniportal endoscopy demand high-precision visualization to minimize complications such as dural tears), is accelerating the adoption of edge computing architectures to process imaging data locally [187]. Crucially, the integration of high-throughput sequencing data, especially RNA-sequencing profiles of inflammatory or matrix-remodeling genes from affected tissues, allows for the direct correlation of imaging-derived biomarkers with underlying molecular pathways, dramatically enhancing diagnostic specificity and mechanistic understanding [188]. Ultrasound elastography further complements this by monitoring synovial inflammation through quantitative stiffness mapping and guiding interventions like corticosteroid injections with greater precision [189]. However, physicians face considerable challenges in the broad clinical translation of these advanced approaches, including the nuance of integrating heterogeneous data from disparate imaging, omics, and clinical platforms, the urgent need for robust standardization of acquisition protocols and analytical pipelines to ensure interoperability and reproducibility, and the validation of these sophisticated tools across diverse patient populations to confirm clinical utility beyond specialized academic centers [190]. Overcoming these hurdles, potentially through the development of interoperable, cloud-based informatics platforms that streamline data workflows and facilitate real-time access to integrated imaging and sequencing information, is critical for realizing the full potential of image-guided molecular profiling as a vital component of precision musculoskeletal care, delivering truly personalized diagnostics and interventions for degenerative joint diseases [191].

In addition to image-guided profiling, predictive modeling holds significant promise in providing personalized treatment.

### 8.2. Predictive Modeling of Tissue Remodeling

Predictive modeling is revolutionizing musculoskeletal diagnostics by employing sophisticated computational architectures to forecast tissue remodeling dynamics and inform personalized therapeutic stratification, a domain primed for innovation by software engineers adept at constructing intricate, data-driven systems [192]. Advanced algorithmic frameworks, such as ensemble learning via Random Forest classifiers, integrate high-dimensional radiomic signatures extracted from modalities like qCT or MRI using texture analysis and geometric quantification with multi-omics data, such as transcriptomic profiles from RNA-sequencing that delineate aberrant gene expression patterns fundamental to tissue pathology. In doing so, prognostication of cartilage degradation with feature importance is to be rigorously assessed through metrics like Gini impurity or permutation-based scores [193]. Concurrently, Bayesian networks provide a robust probabilistic graphical modeling approach for dissecting complex processes such as bone mineralization kinetics, capably incorporating biophysical parameters (e.g., molecular dynamics-derived ligand-receptor binding affinities) and inherently propagating uncertainty through predictive inference, a critical capability for bolstering clinical decision support [194]. Furthermore, Graph Neural Networks (GNNs) leverage graph convolutional layers and attention mechanisms to exceptionally decipher the intricate relational topology of synovial cytokine signaling networks, which is often elucidated through system-level analysis of gene expression programs governing immune cell activation and intercellular communication. This allows for the subsequent prediction of inflammatory progression by learning latent representations of molecular and cellular entities and their dynamic interactions [195]. These cutting-edge models are meticulously engineered to ingest and synergistically process multi-modal inputs, thereby capturing the non-linear, complex spatiotemporal trajectories characteristic of degenerative joint diseases [196]. The development pipelines for such predictive systems necessitate rigorous data pre-processing, encompassing advanced feature engineering, robust dimensionality reduction techniques (e.g., Principal Component Analysis, Uniform Manifold Approximation and Projection (UMAP), or variational autoencoders). This is followed by meticulous normalization and harmonization strategies to mitigate batch effects and eventually model training on extensively curated, longitudinal datasets essential for capturing the intricate dynamics of tissue adaptation, degeneration, and therapeutic response over time [197]. Indispensable to their clinical translation are robust validation protocols, including stratified k-fold cross-validation and, ideally, prospective validation on independent, external patient cohorts to ensure generalizability, coupled with comprehensive uncertainty quantification. This can be achieved through sophisticated methods like Monte Carlo dropout in deep neural networks, Bayesian inference, or conformal prediction, which is paramount for assessing the reliability of predictions and developing physician confidence when confronted with outputs from these complex algorithmic systems [198]. The profound integration of imaging-derived biomarkers, such as qCT-derived trabecular connectivity indices or elastography-quantified synovial stiffness reflecting alterations in extracellular matrix integrity due to modified cellular biosynthetic programs, with granular molecular data from high-throughput sources like single-cell RNA-sequencing (scRNA-seq), which illuminates cellular heterogeneity, identifies rare pathogenic cell populations, and maps cell-specific gene expression landscapes, endows these predictive models with enhanced biological plausibility and unparalleled predictive specificity [199]. Consequently, these sophisticated predictive tools can substantively guide critical clinical decisions, including the identification of optimal intervention windows for disease-modifying therapies like bisphosphonates by forecasting bone mineral density trajectories or for anti-cytokine biologics by predicting inflammatory flare-ups in synovium-based on modeled cytokine network activity intrinsically linked to specific pathogenic gene activation patterns [200]. In cartilage research, computational models predicting Matrix MetalloProteinase (MMP)-driven degradation, where specific MMP gene upregulation is a key indicator of catabolic activity, directly inform the rational design and optimization of targeted inhibitor therapies, while in bone tissue engineering, such models refine the micro-architectural design and material composition of regenerative scaffolds by simulating cellular responses and tissue integration [201]. Despite their transformative potential, significant translational hurdles persist, including the intense computational demands necessitating access to distributed high-performance computing clusters equipped with GPUs/TPUs; the formidable challenges of managing, harmonizing, and imputing meaning from highly heterogeneous, often sparse, and multi-scale data; and the crucial, frequently physician-voiced, imperative for model interpretability, now increasingly addressed by emerging eXplainable AI (XAI) techniques (e.g., SHAP, LIME, attention mapping) designed to de-mystify “black-box” algorithms and thereby foster clinical trust, facilitate regulatory approval, and ensure seamless integration into established clinical workflows [202]. Advanced development platforms such as TensorFlow (2nd generation), PyTorch, and specialized bioinformatics environments (e.g., Bioconductor, scikit-learn) are pivotal in streamlining the creation, validation, and deployment of these sophisticated models, fostering a vibrant ecosystem of innovation, particularly prominent in technology-driven regions like the San Francisco Bay Area, thereby firmly positioning predictive modeling as a central tenet of next-generation musculoskeletal diagnostics aimed at achieving early, precise disease detection and truly personalized therapeutic strategies [203].

To ensure effective translation into a clinical setting, these tools must be optimized via robust integration into existing healthcare systems.

### 8.3. Clinical Integration and Workflow Optimization

In order to implement these tools at the bedside, hospitals need a robust IT ecosystem that integrates multi-modal imaging (via PACS enhanced with DICOMweb/HL7 and EHRs via SMART on FHIR interfaces) with AI-driven Clinical Decision Support Systems (CDSS) for automated biomarker quantification and risk stratification, alongside high-throughput sequencing data [204]. Workflow optimization leverages cloud architectures (e.g., AWS, Azure, GCP) and containerized, automated pipelines (e.g., MONAI, Nextflow, TensorFlow Serving) for standardized image processing, machine learning model deployment and multi-omics (e.g., spatial transcriptomics, scRNA-seq) data integration, thereby correlating imaging-derived endotypes with granular molecular signatures to inform precision interventions like qCT-guided bone graft sizing, MRI-guided stem cell delivery, or ultrasound-elastography-informed biologics administration [205]. Despite significant advancements, persistent challenges include navigating stringent regulatory landscapes (e.g., FDA clearance for AI), ensuring data privacy and security (HIPAA/GDPR compliance, robust encryption, RBAC, MFA, audit logging), mitigating algorithmic bias with fairness-aware machine learning, and scaling solutions like edge computing for real-time, point-of-care analytics, all of which underscore the need for incorporating computational imaging, data science, and AI literacy into medical education through simulation-based training and interdisciplinary curricula to fully realize data-driven, personalized care.

## 9. Discussion

In this review, we show that the therapeutic landscape for degenerative joint diseases is rapidly changing, as molecule-level analysis can now be integrated with imaging modalities in order to give clinicians a more complete picture of patient health. We have detailed how advanced imaging modalities—including high-resolution MRI providing unparalleled soft-tissue contrast and biomolecular sensitivity (e.g., dGEMRIC, T1ρ, ^23^Na MRI), qCT offering precise bone microarchitecture quantification, DECT enabling material decomposition and inflammation mapping, ultrasound elastography assessing tissue biomechanics, and vibroarthrography determining cartilage integrity and contact mechanics—are generating rich, multi-parametric datasets [206]. These imaging techniques, however, realize their full potential only when synergistically integrated with sophisticated computational frameworks and high-throughput sequencing technologies [108,109,110,111,112,113,114,115,116,117,118,119,120,121,122,123,124,125,126,127,128,129,130,131,132].

Radiomic feature extraction and deep learning pipelines, particularly leveraging architectures like CNNs (e.g., U-Net, ResNet) and increasingly GNNs, are automating complex image analysis tasks, enhancing segmentation accuracy, and enabling the discovery of sub-visual imaging biomarkers [207]. Compositional MRI techniques such as T_2_ and T_1_ρ mapping have been the subject of multiple systematic reviews and meta-analyses and have proven to have discriminative validity in identifying early cartilage changes in individuals at risk of knee OA [208]. Similarly, DECT and virtual non-calcium imaging, in particular, have been shown in recent meta-analyses to possess high diagnostic performance for detecting bone marrow lesions in knee OA, with specificities exceeding 90% and excellent AUC values [209]. AI-augmented segmentation, refined by attention mechanisms and informed by structural predictions from tools like AlphaFold2, is also becoming crucial for precise ROI delineation. Multi-scale modeling, integrating data from molecular dynamics simulations, agent-based models, and finite element analyses driven by platforms like COMSOL Multiphysics or OpenCMISS, allows physicians to simulate complex biological processes from molecular interactions to tissue-level responses. COMSOL Multiphysics (version 5.3a, Burlington, MA, USA), in particular, is well-suited for finite-element analysis of complex physiological systems, allowing for the simulation of stress distributions and cartilage deformation under load [210]. The molecular context is provided by omics data: transcriptomics (RNA-seq, scRNA-seq, spatial transcriptomics using platforms like 10x Visium and analysis tools like STAR, DESeq2, Seurat), proteomics (mass spectrometry via Orbitrap Fusion, analyzed with MaxQuant, MixOmics), and potentially other omics layers. This is then correlated with imaging-derived features, protein interaction networks, and cellular pathway dysregulations (e.g., MMP activity, cytokine signaling like IL-6/TNF-α/NF-κB/JAK-STAT, DNA repair pathways like UBE2T-FANCL) [147,148,149,150,151,152,153,154,155,156,157,158,159,160,161,162,163,164,165,166,167,168,169,170,171,172,173,174,175,176,177,178,179,180,181].

In addition, reproducibility and validation remain significant barriers to enabling radiomics and AI to be applied in musculoskeletal evaluation. Given that many models rely on limited or single-center datasets, results may not be generalizable [211]. A recent systematic review of CT and MRI radiomics in bone and soft-tissue sarcomas shows that only about 59 % of studies assessed feature reproducibility, primarily through test–retest scans, and just 62 % employed internal cross-validation, with an even lower 25% including external validation sets [212]. Therefore, it is imperative to establish standardized imaging and omics repositories across numerous institutions. Moreover, with the limited interpretability of deep learning models, due to factors such as high-dimensional radiomic feature spaces combined with small sample sizes, validation challenges remain pertinent potentially hindering clinical decision-making [213]. To address these issues, further work needs to be done in developing explainable AI systems and creating publicly available benchmark datasets that incorporate diverse patient populations. Furthermore, incorporating these advancements into medical education through simulation-based training and interdisciplinary curricula is crucial for equipping future clinicians with the tools to succeed [214].

## 10. Conclusions

### 10.1. Clinical Implications

In all, it is our belief that this multimodal data fusion is paving the way for transformative, translational applications. When integrated, these modalities will enable physicians to evaluate molecular tissue alterations with unseen depth and resolution. Image-guided molecular profiling can soon enable precise therapeutic interventions, predictive modeling (employing Random Forest, Bayesian networks, and GNNs trained on platforms like TensorFlow or PyTorch) forecasts disease trajectories and treatment responses, merging diagnostics with nanoparticle-based targeted therapy (e.g., SPIOs, liposomes, gold nanoshells), promises real-time treatment optimization and monitoring. However, the clinical actualization of these powerful tools hinges on robust IT infrastructure and optimized workflows. Streamlined integration into PACS and EHR systems via SMART on FHIR interfaces, supported by cloud-based platforms (AWS, Azure, GCP) for scalable storage and computation, and automated, containerized pipelines (using MONAI, Nextflow, Docker, Kubernetes) are essential. Addressing challenges such as data privacy (HIPAA, GDPR compliance), security (encryption, MFA, audit trails), regulatory approval for AI algorithms (FDA clearance), data heterogeneity, algorithmic bias, and the need for model interpretability (XAI techniques like SHAP, LIME) remains paramount. Thus, by synergistically applying imaging, computational analytics, and sequencing technologies, clinicians can understand the various players that induce joint diseases.

### 10.2. Future Directions

Despite these advances, widespread clinical adoption faces numerous challenges related to reproducibility, model explainability, limited multi-institutional datasets, and the need for standardization across varying patient populations. By overcoming the existing technical and translational hurdles and fostering a data-driven, integrated approach, the medical community can significantly advance diagnostic accuracy, enable early detection, and deliver truly personalized and predictive interventions, thereby revolutionizing musculoskeletal care and fulfilling the promise of precision medicine for patients suffering from these debilitating conditions. By extending beyond traditional single-modality reviews and incorporating the newest advances in quantitative imaging, this work bridges established principles with emerging precision tools in musculoskeletal care. This integrated vision directly supports the evolving landscape of innovative imaging and diagnostic technologies, driving forward the objectives of enhanced patient outcomes through technological convergence.

### 10.3. Key Takeaways

- Multimodal integration of imaging and computational technologies enables molecular-level evaluation of musculoskeletal systems with significant depth.
- Advanced imaging modalities, particularly MRI, qCT, DECT, ultrasound elastography, and vibroarthrography provide complementary structural and functional insights.
- Radiomics and deep learning models (e.g., CNNs, U-Net, GNNs) enhance tissue characterization and are able to predict disease progression with increasing accuracy.
- Transcriptomic and proteomic integration with imaging data supports the identification of tissue-specific biomarkers and offers mechanistic insight into joint degeneration.
- Clinical translation is limited by challenges in reproducibility and explainability of AI.
- Future directions should emphasize explainable AI and cross-platform coordination for the incorporation of these technologies into clinical workflows and education.

## Data Availability

No new data were created or analyzed in this study. Data sharing is not applicable to this article.

## Abbreviations

**Abbreviation**	**Full Term**
MRI	Magnetic Resonance Imaging
qCT	Quantitative Computed Tomography
DECT	Dual-Energy Computed Tomography
RNA-seq	RNA Sequencing
GAG	Glycosaminoglycan
MMP	Matrix Metalloproteinase
AI	Artificial Intelligence
ML	Machine Learning
CNN	Convolutional Neural Network
T1ρ	T1 Rho (Spin-lattice relaxation in the rotating frame)
